# Effect of Crocin From Saffron (*Crocus sativus* L.) Supplementation on Oxidant/Antioxidant Markers, Exercise Capacity, and Pulmonary Function Tests in COPD Patients: A Randomized, Double-Blind, Placebo-Controlled Trial

**DOI:** 10.3389/fphar.2022.884710

**Published:** 2022-04-20

**Authors:** Hassan Ghobadi, Nasim Abdollahi, Hanieh Madani, Mohammad Reza Aslani

**Affiliations:** ^1^ Lung Diseases Research Center, Ardabil University of Medical Sciences, Ardabil, Iran; ^2^ Department of Internal Medicine, Faculty of Medicine, Ardabil University of Medical Sciences, Ardabil, Iran; ^3^ Faculty of Medicine, Ardabil University of Medical Sciences, Ardabil, Iran; ^4^ Applied Biomedical Research Center, Mashhad University of Medical Sciences, Mashhad, Iran

**Keywords:** crocin, oxidative stress, COPD, 6MWD (6minute walking distance), NF-kB

## Abstract

**Background:** Chronic obstructive pulmonary disease (COPD) is a progressive and chronic respiratory disorder characterized by reversible airflow limitation and lung parenchyma destruction. The main feature of COPD is inflammation and disturbance of the oxidant/antioxidant balance in the airways. The therapeutic use of herbal supplements with antioxidant and anti-inflammatory properties seems to be very useful in the medical management of patients with COPD.

**Method:** COPD patients were divided into placebo and intervention groups (each group n = 23) in a clinical trial study. The intervention group received crocin supplementation (30 mg/day for 12 weeks), and the control group received a placebo. Pre- and after the intervention, pulmonary function tests (PFTs), exercise capacity (using a 6-min walking distance test (6MWD)), and serum levels of total oxidant status (TOS), total antioxidant capacity (TAOC), and NF-kB were assessed using the ELISA test.

**Results:** Intervention with crocin for 12 weeks in COPD patients decreased serum levels of TOS and NF-κB as well as increased TAOC. In addition, the results of the 6MWD test reveal an improvement in patients’ exercise capacity.

**Conclusion:** Crocin supplementation appears to effectively establish oxidant/antioxidant balance and improve inflammatory conditions in patients with COPD.

Iranian Registration of Clinical Trials No: IRCT20110109005579N2.

## Introduction

Chronic obstructive pulmonary disease (COPD) is a progressive and chronic respiratory disorder characterized by reversible airflow limitation and destruction of lung parenchyma (Decramer et al., 2012). Various factors are involved in the development and progression of COPD, such as sex, age, genetic factors, chronic bronchitis, exposure to particles, and infection (Halpin et al., 2021). Smoking is a predominant risk factor in patients with COPD, as it contains various harmful substances that stimulate reactive oxygen species (ROS) production and induce oxidative damage (Bernardo et al., 2015). Although the pathophysiological mechanism of COPD has not been well clarified, certain factors have been reported, including neutrophil airway inflammation, oxidative stress, protease–antiprotease imbalance, and apoptosis (Amani et al., 2017; Barnes, 2017; Ghobadi et al., 2017).

Oxidative stress is caused by an imbalance in ROS-induced oxidants and endogenous antioxidants (Marotta et al., 2011). Environmental sources for ROS production include cigarette smoking, car exhaust fumes, industrial pollution, and occupational exposure to dust (Antunes et al., 2021). In contrast, cellular sources for ROS production include activation of xanthine oxidase (XO) and nicotine adenine disphosphonucleotide (NADPH) oxidase (Taniguchi et al., 2021). Inflammation of the airways is closely related to oxidative stress processes in COPD patients. ROS-induced airway inflammation leads to the recruitment of inflammatory cells to the airways and the production of pro-inflammatory cytokines, which increase the severity of inflammation and increase ROS production (Kirkham and Barnes, 2013). Transcription factors such as nuclear factor kappa B (NF-kB) play a significant role in the interaction between inflammation and ROS in chronic inflammatory diseases (Mahmoud et al., 2021). Therefore, one of the critical therapeutic targets of COPD patients is the management of inflammation and oxidative stress.

Therapeutic use of some foods and herbs has been of interest to humans throughout history for the prevention and treatment of diseases and health problems (Boskabady et al., 2007; Ghasemi et al., 2021; Khazdair et al., 2021; Saadat et al., 2021). Saffron (Crocus sativus L.) is a valuable plant used as a food additive and medicinal plant (Boskabady and Aslani, 2006). Therapeutic effects of saffron have been reported in various disorders such as cardiovascular, asthma, diabetes, and autoimmune (Hashemzaei et al., 2020; Aslani et al., 2021). Major compounds of saffron include crocin, crocetin, safranal and picrocrocin (Kermani et al., 2017a; Saadat et al., 2019). The antioxidant and anti-inflammatory properties of crocin have been proven in animal and human studies (Kermani et al., 2017a; Aslani et al., 2021). In chronic inflammatory diseases such as diabetes and asthma, clinical studies have reported the protective role of saffron in reducing inflammatory markers (Hosseini et al., 2018; Shahbazian et al., 2019). Recently, Krocina™ tablets, a 98% purified crocin from saffron, have been used in clinical trial studies (Poursamimi et al., 2020). The anti-inflammatory effects of Krocina™ have been identified in patients with osteoarthritis by reducing C-reactive protein (CRP) and interleukin (IL)-17 levels (Poursamimi et al., 2020).

Accordingly, the primary aim of the current study was to evaluate the effects of 12 weeks of crocin from saffron intervention on oxidant/antioxidant and inflammatory markers in COPD patients. Secondary objectives were determined for exercises capacity and pulmonary function tests (PFTs).

## Subjects and Methods

### Design

The current study was a randomized, double-blind, placebo-controlled clinical trial.

### Participants

The study was carried out in 2020 at Ardabil Imam Khomeini Hospital in northwestern Iran. Male patients with COPD were eligible to participate in the study by having the following conditions: one- clinical criteria such as shortness of breath, sputum, and cough, and two- spirometric findings (Forced expiratory volume (FEV1) <80% and FEV1/Forced vital capacity (FVC) < 70%). Exclusion criteria were one- hospitalization history during the last 3 months, two- any lung disease other than COPD, three- infectious diseases, 4-rheumatoid arthritis, 5-cancer, 6- history of drug use other than COPD-related drugs, and 7- patients with structured physical activity or planned exercise. Exclusion criteria were selected based on previous clinical trial studies that may have influenced the results of oxidant/antioxidant factors, such as supplements and medications, mentioned inflammatory diseases, and daily physical activity.

### Randomization

Using a practical sampling methodology, patients were included in the study and randomized into two groups (placebo and intervention, n = 23). In order to randomize patients, the RANDBETWEEN command was done in Excel. A placebo and intervention tablets were placed in numbered bags by one of the investigators who did not participate in the study. Numbered bags were assigned to participants unaware of random sequences by another researcher. Random codes blinded all study subjects (both researchers and patients).

### Intervention

After obtaining permission from the university authorities in the intervention group, the samples that were eligible for entering the study were included in the study. The intervention group was given crocin at a 30 mg/day concentration for 12 weeks (Talaei et al., 2015), while the control group received a placebo with the same form and concentration of the drug. Crocin and placebo tablets were prepared by Sina Pooyesh Drug Company (www.samisaz.com, Registration Number 486769) (Poursamimi et al., 2020). Participants were recommended to avoid fast foods, saffron, sausages, and canned foods during the study.

### Outcomes and Relevant Measures

The primary outcome was determined by serum levels of oxidant/antioxidant markers and NF-kB, and the secondary outcome was pulmonary function tests and a 6-min walking distance test (6MWD) test.

### Demographic and Clinical Assessments Questionnaire

Demographic information on age, height, and weight was completed for everyone. Body mass index (BMI) at the beginning and the end of the study was calculated based on height and weight. The pulmonary function test (including FEV1, FVC, and FEV1/FVC) and the 6MWD test were also evaluated before and after the intervention.

### Biochemical Examinations

At the beginning and end of the study, blood samples were taken from patients to evaluate serum levels of total antioxidant capacity (TAOC), total oxidant status (TOS), and NF-kB. The ELISA technique and commercial kits (Crystal day, China) were used to determine TAOC, TOS, and NF-kB serum levels.

### Sample Size Estimation

**FIGURE 1:**
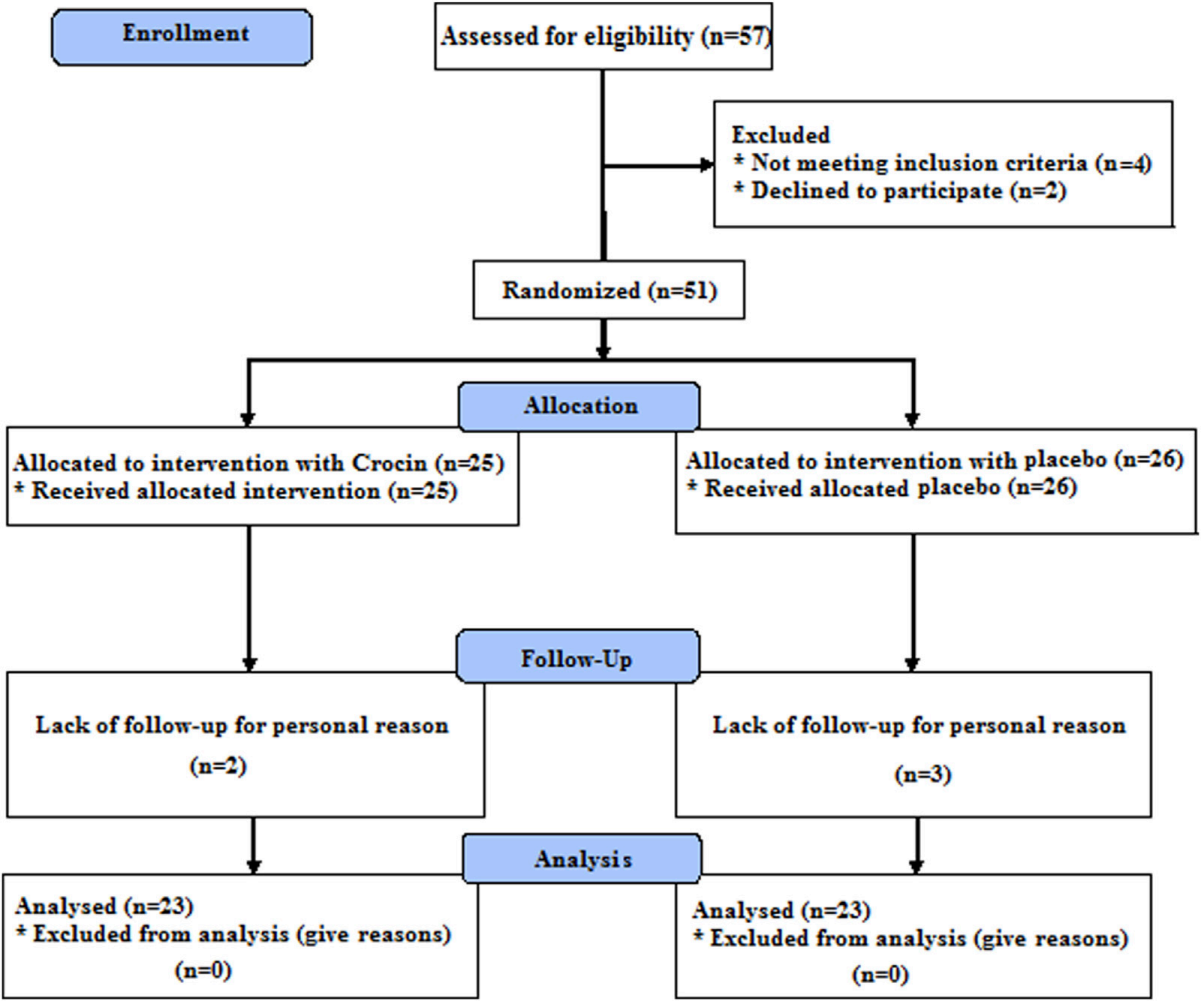
Flow diagram of the trial.

According to the previous study, the sample size in this study was estimated at 22 subjects, of which 25 individuals were included in each group (Hosseini et al., 2018). Twenty-three individuals in each group completed the study (Figure 1).

### Ethical Considerations

The current study was approved by the Human Ethics Committee of Ardabil University of Medical Sciences with an ethics code: IR. ARUMS.REC.1398.428 and was registered in the Iranian Registration of Clinical Trials No.: IRCT20110109005579N2. Informed written consent was obtained from all individuals. All patients were free to leave the study at any study stage.

### Statistical Analysis

The normal distribution of the data was determined from the Kolmogorov-Smirnov test. Parametric data were reported using the mean ± standard deviation (SD), and non-parametric data were reported using the 25th-75th percentiles. Paired *t*-test (parametric) and Wilcoxon (non-parametric) tests were used to analyze each group’s data before and after the intervention. Independent t-tests and Mann-Whitney tests were used for comparing data between placebo and intervention groups. *p* < 0.05 was defined as statistically significant. SPSS version 21 and Graph Pad Prism 7 software were used for the statistical analysis.

## Results

### Characteristics of Subjects

The parameters considered in the current study are presented in Table 1. There was no significant difference between the placebo and intervention groups at the beginning of the study regarding age, BMI, FEV1, FVC, FEV1/FVC, 6MWD, and serum levels of TAOC, TOS, and NF-kB variables.

**TABLE 1:** Baseline parameters in the study groups.

Variables	Placebo (n = 23)	Crocin (n = 23)	*p*-value
	Baseline	Baseline	
Age (year)	61.72 ± 8.54	62.04 ± 8.83	0.904
Weight (kg)	68.81 ± 12.12	74.82 ± 10.99	0.089
Height (m^2^)	1.71 ± 0.05	1.72 ± 0.05	0.842
BMI (kg/m^2^)	23.18 ± 4.31	25.13 ± 3.62	0.109
FEV1 (%)	58.26 ± 15.95	55.39 ± 13.91	0.519
FVC (%)	76.21 ± 15.85	70.34 ± 14.92	0.203
FEV1/FVC ratio	62.23 ± 9.82	64.67 ± 9.25	0.391
6MWD (m/min)	380.68 ± 113.64	397.61 ± 64.91	0.546
TAOC (ng/ml)	2.48 ± 0.60	2.57 ± 1.49	0.787
TOS (ng/ml)	4.87 ± 1.61	5.04 ± 0.82	0.661
NF-kB (ng/ml)	4.57 ± 1.27	4.79 ± 1.04	0.543

BMI: body mass index, FEV1: forced expiratory volume in the first second, FVC: forced vital capacity, 6MWD: 6-min walking distance test, IL-6: interleukin-6, TNF-α: tumor necrosis factor alpha.

### Effects of Crocin-Intervention on PFTs and 6MWD

**FIGURE 2:**
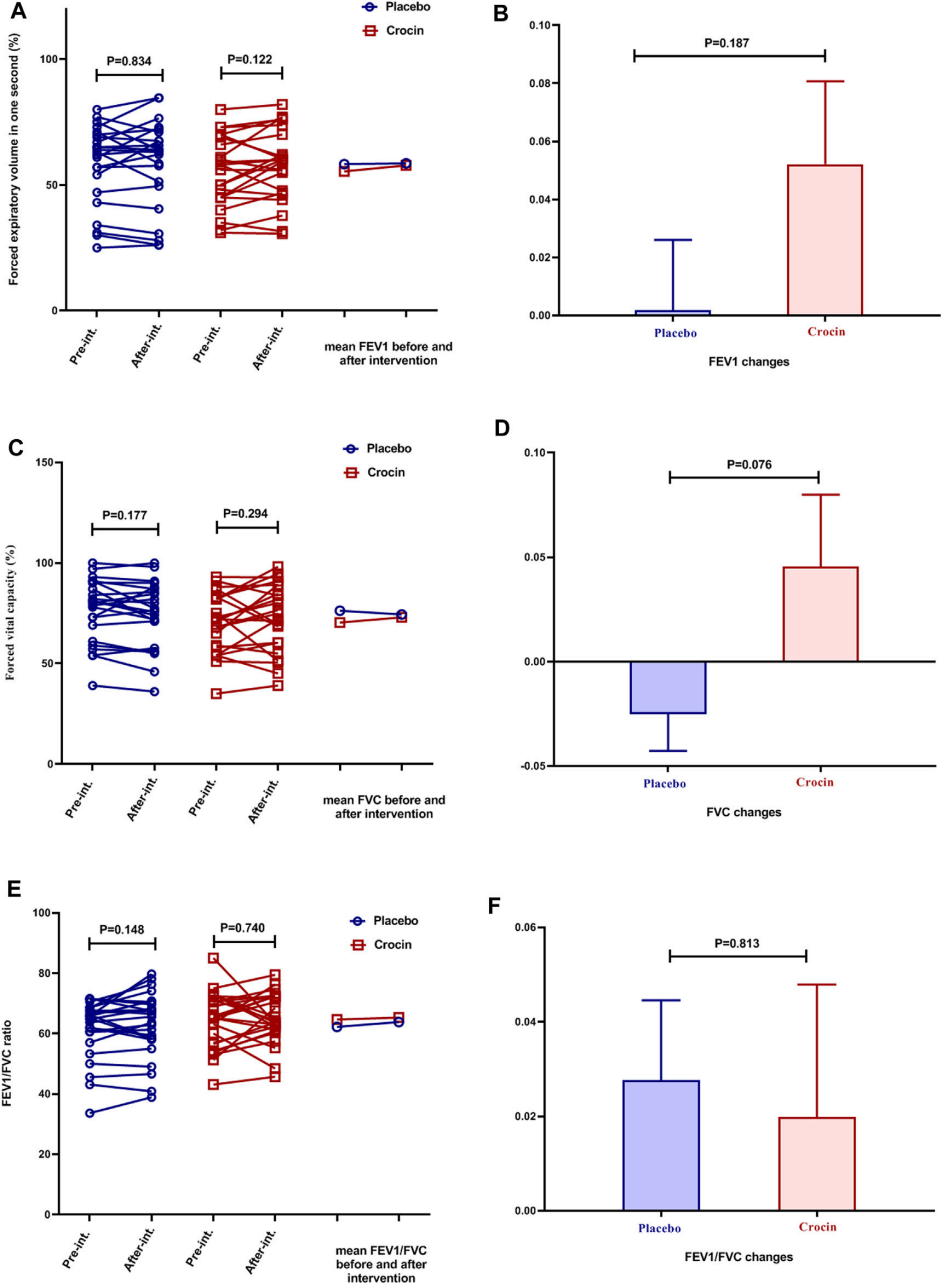
Individual values and mean of **(A)**: FEV1 **(B)**: FEV1 changes **(C)** FVC **(D)**: FVC changes **(E)**: FVE1/FVC, and **(F)**: FEV1/FVC changes in placebo (blue color) and crocin-treated (red color) group’s pre-intervention and after 12 weeks of intervention. FEV1: Forced expiratory volume in the first second, FVC: Forced vital capacity.

In both placebo and Crocin-treated groups, it was found that there was no significant difference in the mean FEV1, FVC, and FEV1/FVC ratio (Figures 2A,C,E). In addition, the mean changes of FEV1, FVC, and FEV1/FVC ratio also did not reveal a significant difference between the two groups (Figures 2B,D,F).

**FIGURE 3:**
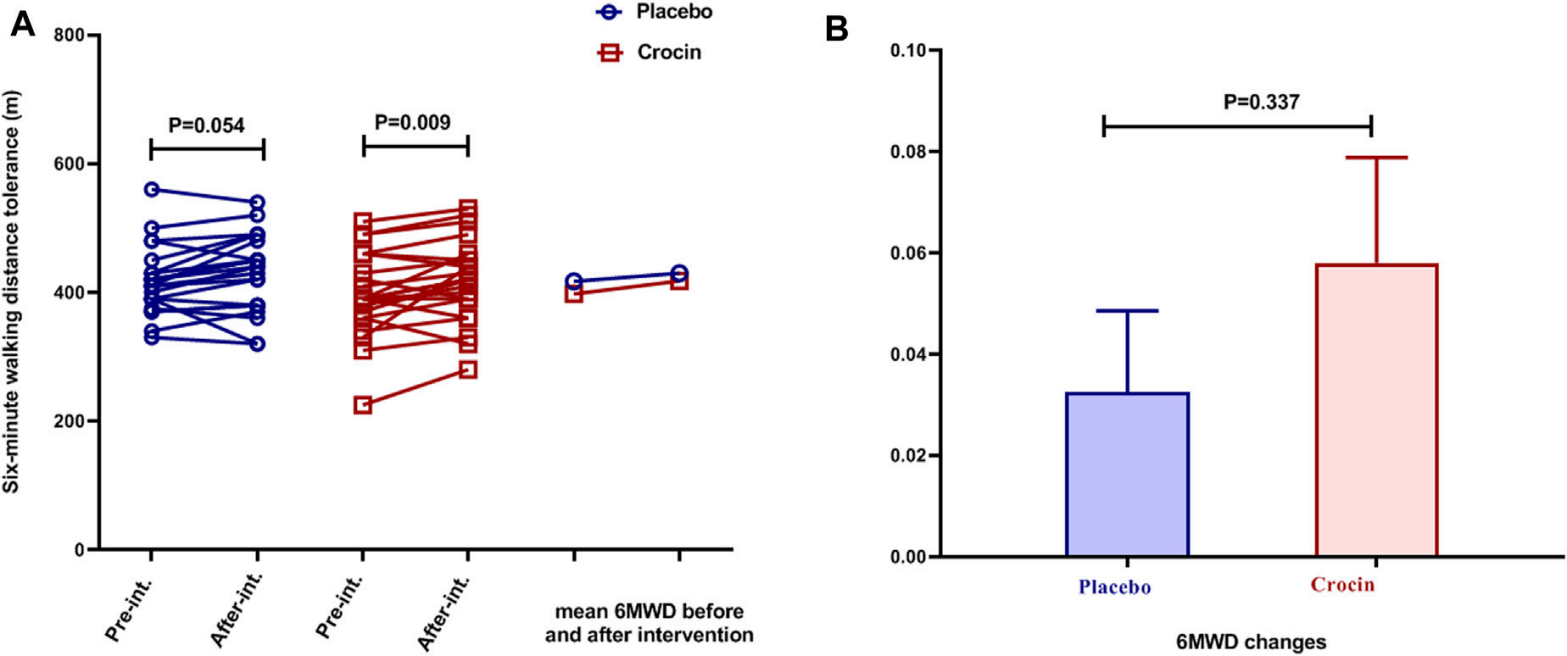
Individual values and mean of **(A)**: 6MWD and **(B)**: 6MWD changes in placebo (blue color) and crocin-treated groups (red color) pre-intervention and after 12 weeks of intervention. 6MWD: 6-min walking distance test.

In the placebo group, despite the increase in mean 6MWD at the end of the study, no significant difference was observed, while in the intervention group, a significant increase was evident (*p* < 0.01, Figure 3A). Mean changes of 6MWD were not significantly different between the placebo and intervention groups (Figure 3B).

### Effects of Crocin-Intervention on Serum TOS, TAOC, and NF-kB Levels

**FIGURE 4:**
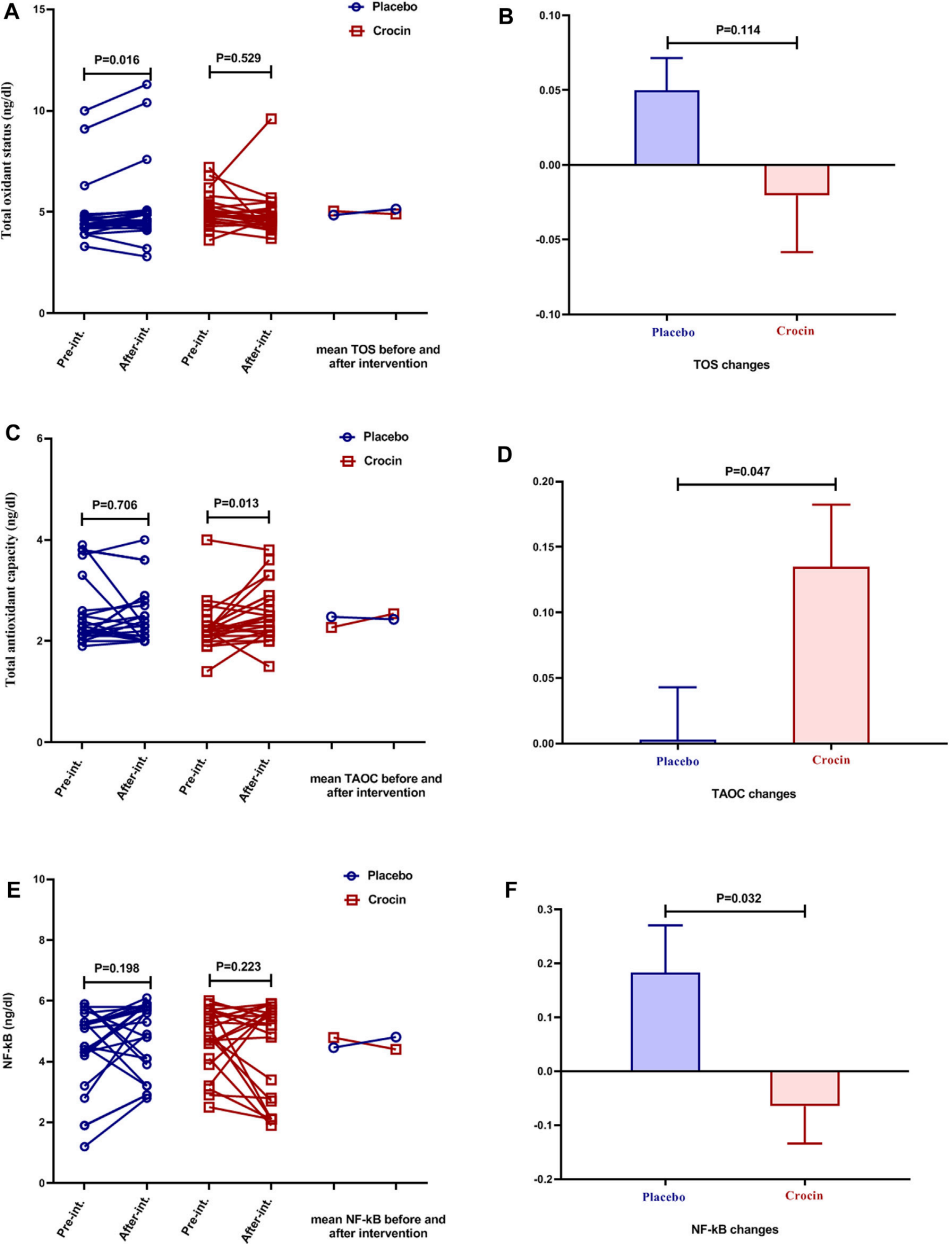
Individual values and mean of serum levels of **(A)**: TOS **(B)**: TOS changes **(C)**: TAOC **(D)**: TAOC changes **(E)**: NF-kB, and **(F)**: NF-kB changes in placebo (blue color) and crocin-treated groups (red color) pre-intervention and after 12 weeks of intervention. TOS: total oxidant status, TAOC: total antioxidant capacity.

Significantly increased levels of TOS were seen in the placebo group at the end of the study compared to the beginning of the study (*p* < 0.05, Figure 4A), but there was no significant difference in the intervention group. The mean TOS changes between the placebo and Crocin-treated groups were insignificant (Figure 4B).

After the intervention, the mean serum TAOC levels in the intervention group were significantly higher than in pre-intervention (*p* < 0.05, Figure 4C), but no significant differences were observed in the placebo group. Furthermore, the mean changes in serum TAOC concentrations were significantly higher in the intervention group than in the placebo group (*p* < 0.05, Figure 4D).

Serum NF-κB levels were not significantly different in the placebo and intervention groups after the intervention than pre-intervention (Figure 4E). However, the results of mean changes in serum NF-κB levels showed a significant decrease in the Crocin-treated group compared to the placebo group (*p* < 0.05, Figure 4F).

### Side Effects

No drug side effects were observed in individuals receiving the crocin intervention. Individuals who withdrew from the study for personal reasons and the study coincided with the COVID-19 pandemic.

## Discussion

The most important results of the current clinical trial study were: one- decreased serum TOS and NF-kB levels, two- increased serum TAOC levels, and three- improved 6MWD tolerance.

COPD is an inflammatory disease characterized by the involvement of the lung parenchyma, airways, and pulmonary vasculature (Halpin et al., 2021). The pathophysiology of COPD is thought to be involved in oxidant/antioxidant and protease/antiprotease imbalances (Boukhenouna et al., 2018). As a result of increased activity of oxidants and proteases, destruction of air sacs has been reported in patients with COPD (Boukhenouna et al., 2018). PFT changes are critical diagnostic, grading and monitoring criteria for COPD patients (Halpin et al., 2021). There is evidence of a decrease in FEV1 in COPD patients due to inflammatory responses and airway obstruction. The intervention with Crocin showed that FEV1 in COPD patients was enhanced, although it was not significant. One of the factors that may have influenced the PFT results of the current study is the duration of the intervention for 3 months. The effectiveness of most supplements has been reported in clinical trial studies with more than 3 months of intervention. Hosseini et al. demonstrated that saffron intervention improves pulmonary function tests in asthmatic patients (Hosseini et al., 2018). Although the exact mechanism of saffron and its active ingredient (Crocin) is poorly understood, its anti-inflammatory and antioxidant effects may play a key role (Kermani et al., 2017b). Most animal studies have shown the protective effects of saffron and Crocin in the ovalbumin-induced asthma model (Saadat et al., 2019; Aslani et al., 2021). However, further human studies are required to elucidate the effects of Crocin on PFTs in chronic lung diseases.

Oxidative stress is a critical factor in promoting COPD inflammation (Zinellu et al., 2021). Oxidative stress occurs when endogenous antioxidant defenses are impaired, or reactive oxygen species (ROS) activity is enhanced (Kirkham and Barnes, 2013). ROS production originates from environmental (cigarette smoke) or cellular (inflammatory and structural cells) sources (Kirkham and Barnes, 2013). Pulmonary inflammation induced by ROS leads to the production of pro-inflammatory markers and the recruitment of inflammatory cells into the airways, thereby increasing the production of ROS (Zinellu et al., 2021). In patients with COPD, oxidative stress is typically caused by prolonged exposure to cigarettes smoke or by a variety of inflammatory and immune stimuli in the airways (Taniguchi et al., 2021). Elevated levels of oxidants and decreased antioxidant levels are present locally (in lung tissue) and systemically in patients with COPD (Elmasry et al., 2015). Patients with COPD showed increased levels of oxidative products such as malonyl dialdehyde (MDA) and TOS compared to healthy subjects (Zeng et al., 2013). Decreased antioxidants may also contribute to increased oxidative stress in COPD conditions, such as SOD, GSH-Px, reduced GSH, TAOC, thioredoxin, and nuclear factor erythroid two– related factor 2 (Nrf2) (Taniguchi et al., 2021).

The study showed that Crocin treatment in COPD patients caused a significant reduction in serum TOS levels. On the other hand, Crocin treatment also increased TAOC levels in COPD patients. In fact, the results suggest that Crocin treatment reversed the oxidant/antioxidant imbalance created by COPD. Saffron has potent anti-inflammatory and antioxidant effects with various components such as Crocin, crocetin, and safranal (Assimopoulou et al., 2005). Invitro, *in vivo*, and human studies have shown the antioxidant effects of saffron and Crocin in various pathological conditions, including asthma, COPD, myocardial infarction, and cancer (Tsantarliotou et al., 2013; Al-Gubory, 2014). The saffron and its effective compounds exert their antioxidant effects by reducing the production of oxidative factors such as MDA, lipid peroxidation (LPO), inducible nitric oxide synthase (iNOS), nitric oxide (NO), XO, myeloperoxidase (MPO), and protein carbonyls (PC) as well as by increasing antioxidant factors such as reduced glutathione (GSH), total antioxidant capacity (TAOC), superoxide dismutase (SOD), glutathione peroxidase (GPx), catalase (CAT), and glutathione-S-transferase (GST) (Boskabady and Farkhondeh, 2016).

Different clinical trial studies have examined the effects of saffron on oxidative markers in various diseases. Intervention with saffron in type 2 diabetic patients reduced serum MDA concentrations while no effect was observed on TAOC and F2-isoprostane levels (Azimi et al., 2014; Ebrahimi et al., 2019; Shahbazian et al., 2019). In patients with ulcerative sclerosis, it was revealed that intervention with saffron for 8 weeks resulted in increased TAOC, GPX, and SOD while preventing an increase in serum MDA concentration compared with the placebo group (Tahvilian et al., 2021). Also, in patients with multiple sclerosis, a 4-weeks intervention with saffron decreased MDA levels and increased TAOC (Ghiasian et al., 2019). In addition, similar results occurred in patients with nonalcoholic fatty liver diseases due to intervention with saffron for 12 weeks, decreasing MDA and increasing TAOC levels (Pour et al., 2020). In patients with metabolic syndrome, it has been reported that intervention with saffron for 12 weeks can modulate pro-oxidant-antioxidant serum levels (Kermani et al., 2015). However, in patients with rheumatoid arthritis, Hamidi et al. did not observe significant differences in MDA and TAOC levels after 12 weeks of saffron intervention (Hamidi et al., 2020). For the first time in a clinical trial study on COPD patients, the results of our study reported the reducing effects of Crocin on oxidative factors and the increasing effects of antioxidant factors.

Recently, there has been much evidence that oxidative stress and inflammation play a vital role in the pathogenesis of various diseases, including diabetes, cancer, obesity, metabolic syndrome, and chronic respiratory disease (Mahmoud et al., 2021). By activating inflammatory signaling pathways, ROS causes the release of different inflammatory mediators such as cytokines, chemokines, and eicosanoids (Mahmoud et al., 2021). Activation of some protein kinases and signaling pathways (nuclear factor-kappaB (NF-kB), p38 mitogen-activated protein kinases (MAPK), and protein kinase C) as a result of oxidative stress also has a double effect on inflammatory processes (Canty et al., 1999; Khan et al., 2020). NF-κB activation in patients with COPD occurs in response to inflammatory mediators such as IL-1β and TNF-α or due to activation of Toll-like receptors (TLRs) following bacterial or viral infections (Edwards et al., 2009). The redox pathway regulates NF-κB signaling due to oxidant/antioxidant imbalance in inflammatory diseases of the airways (Rajendrasozhan et al., 2008). In patients with COPD, regulation of NF-κB signaling activity is one of the essential therapeutic criteria, so the therapeutic use of corticosteroids inhibits NF-κB activity and consequently reduces the levels of inflammatory cytokines.

The present study showed that the Crocin intervention had a protective effect on the serum NF-κB concentration since there was a significant increase in the placebo group at the end of the study. Also, the mean changes in serum NF-κB concentration in the placebo group were higher than in the Crocin-treated group. The results revealed that Crocin intervention had inhibitory effects on NF-κB activity in patients with COPD. Similar results have been reported from human and animal studies about the effects of saffron and Crocin on NF-κB levels (Boskabady and Farkhondeh, 2016). Saffron and crocin exert anti-inflammatory and antioxidant effects through various mechanisms, including modulation of phosphoinositide-3-kinase (PI3K)/Akt, protein kinase C (PKC), mitogen-activated protein kinases (MAPK/ERK), Nrf2, NF-κB p65, c-Jun N-terminal kinases (JNK), Ca2+/calmodulin dependent protein kinase 4 (CAMK4), inducible nitric oxide synthase (iNOS), signal transducer and activator of transcription 6 (STAT6), ER-stress markers, and high-mobility group box 1 (HMGB-1) pathways (Kim et al., 2014; Xiong et al., 2015; Yosri et al., 2017; Dianat et al., 2018; Xie et al., 2019; Zhang et al., 2020; Aslani et al., 2021).

One of the most critical health indicators is cardiorespiratory preparations (Oliveira et al., 2016). n exercise test below the maximum 6-min walk-in for patients with COPD is a crucial activity for assessing cardiovascular and respiratory rehabilitation. With this test, patients’ exercise capacity and health-related quality of life (HRQL) can be evaluated (Wouters et al., 2020). COPD patients have reported reduced levels of 6MWD and quality of life compared with healthy individuals (Puhan et al., 2008). Intervention with Crocin for 12 weeks significantly increased 6MWD in patients with COPD. Crocin with anti-inflammatory and antioxidant effects may have led to increased exercise capacity and quality of life satisfaction in patients with COPD, which requires further studies.

This study had some limitations. First, the current study had no female patients, and the extraction results can only be used in men with COPD. Second, the sample size was moderate considering the COVID-19 conditions and may have masked the significant results of crocin. Finally, although most studies have reported duration of ≥12 weeks for saffron (crocin) supplementation, it is advisable to evaluate a more extended intervention with saffron in future studies.

In summary, the association between oxidative stress and inflammation affects patients with COPD, such as decreased pulmonary function tests, exercise capacity, and quality of life. Under COPD conditions, increased oxidative and inflammatory markers (such as TOS and NF-kB) combined with decreased antioxidant factors (TAOC) may have been associated with decreased exercise capacity and PFT. Intervention with Crocin (one of the main compounds of saffron) increased exercise capacity and PFTs of patients with COPD, possibly by modulating oxidant/antioxidant and inflammatory pathways.

## Data Availability

The original contributions presented in the study are included in the article/Supplementary Materials, further inquiries can be directed to the corresponding author.
